# Editorial: Glucocorticoids and cognition: recent advances in understanding their interaction, with a particular focus on clinical applicability for the treating endocrinologist

**DOI:** 10.3389/fendo.2023.1339165

**Published:** 2023-12-12

**Authors:** Ian Louis Ross, Eystein S. Husebye, Michelle Henry

**Affiliations:** ^1^ Division of Endocrinology, University of Cape Town, Cape Town, South Africa; ^2^ Department of Clinical Science, University of Bergen, Bergen, Norway

**Keywords:** glucococorticoids, cognition, stress, Addison’s disease, Cushing’s syndrome

We assembled a compendium of reviews for the Research Topic, *Glucocorticoids and cognition* which the reader should find both interesting and useful. Our focus was to elicit clinical applicability for the treating Endocrinologist.

Throughout our lives we encounter stress of varying degrees and James et al., described stress as a general adaptation syndrome, consisting of an initial shock phase and an alarm reaction, followed by adaptation to the stressor and a phase of exhaustion. It is during the exhaustion phase that an unwanted negative stimulus can impact the individual’s ability to resist the stressor. Notably, stressors may be acute or chronic in presentation. The sympathetic adrenal medullary system and the hypothalamic pituitary adrenal axis are two critical physiological systems, which respond to stressful conditions and address coping. Excessive cortisol concentrations can result in alterations in the structure and function of critical elements of the brain, including the hippocampus, amygdala and prefrontal cortex, and chronically raised cortisol concentrations, following a stress response, alter the ability to recover from adversity. Stress elicits an array of physiological consequences, for example, digestive, gastrointestinal, immunosuppressive and cardiovascular diseases and there is a strong association between exposure to adverse childhood events and the development of chronic disease during adulthood. Additionally, the impact of stress is to some degree affected by the time sensitive intervals in a person’s life, for example, childhood trauma, occurring prior to the onset of 10 years of age.

Chronic stress is highly associated with the development of major depressive disorders and there are associations between stress and neurodegenerative diseases, in particular, dementia. Chronic stress negatively affects glucocorticoid receptor expression in the prefrontal cortex which has been found to result in cognitive dysfunction at least in animal models, whereas it may lead to dendritic expansion in orbitofrontal cortex and basolateral amygdala. Long-term stress, originating in childhood and adolescence, has been associated with impaired cognition. Impaired executive dysfunction has been linked to chronic stress in addition to impaired spatial memory, reduced hand eye coordination and accuracy performing mathematical tasks. Moreover, there have been studies of early life stress, resulting in dementia later in life. All clinicians should be aware that among older adults that there is a link between emotional stress and the development of impaired cognitive performance On the other hand, resilience is the ability of an individual to maintain physical and psychological well-being, despite encountering adversity. Cognitive reserve linked to education may mitigate the effect of stress on cognition.

In the review by Rusch et al., the influence of the microbiome is described in health and disease. The human gut microbiome includes over 232,000 non-redundant genomes, which have critical roles in metabolism, immunity, development, behaviour, and cognition. Evidence indicates that the hypothalamic pituitary adrenal axis, the gut microbiome and cognition are inextricably linked. The stress response for example, the use of antibiotics, and differences in diet may impact on overall cognitive health. The gut microbiome functions as a network between the central nervous system, the autonomic nervous system, and the endocrine system. It is unclear how information is shared with the brain. It is well established however, that the gut microbiome can stimulate peptide hormone release from cells within the gut mucosa, which has a central nervous system effect. Stress mediated by cortisol alters gastrointestinal transit time, the permeability of the intestine and nutrient availability, which downstream impacts on the diversity of the gut microbiome. Several lines of evidence have established the association between gut dysbiosis, autism, anxiety and depression, Alzheimer’s disease, Parkinson’s disease, and schizophrenia. The gut microbiome is a critical element in the development and maintenance of cognition. The most convincing evidence exists that chronic antibiotic utilization in midlife is associated with impaired cognition late in life. The impact of glucocorticoids appears to have a dichotomous action on the microbiome, with some studies indicating worsening cognition and others indicating evidence for improved cognition. Additionally, histamine receptor 2 antagonists through the suppression of endogenous gastric acid and downstream influence on the microbiome have been associated with deterioration in cognition. Intriguingly, probiotics have by contrast demonstrated an improvement in cognition among persons with Alzheimer’s disease, fibromyalgia and irritable bowel syndrome.

In the review by Papakokkinou and Ragnarsson the impact of Cushing’s syndrome on memory, concentration attention and executive function have been elucidated, and disappointingly, these faculties are not universally restored, despite successful treatment for Cushing’s syndrome. It appears that the most profound cognitive disruption in this group of patients includes impaired attention, disrupted visuospatial processing, altered processing speed and difficulties with executive functioning. Functional MRI localised five brain regions that were affected including the posterior cingulate cortex, occipital lobe and/or cerebellum, thalamus, right postcentral gyrus and left prefrontal cortex. The most profound implications for patients with Cushing’s syndrome are the higher propensity to manifest with poorer memory and concentration, attention deficits and apathy. It remains uncertain the extent to which cognitive training may alter the neurocognitive deficits in the presence of a very serious endocrine disorder.

In the review by Harbeck et al., patients with adrenal insufficiency on replacement therapy demonstrated self-reported problems including difficulty with executive function, memory and impaired quality-of-life. It was postulated that some of the neuropsychological and cognition deficits mainly in the domains of attention, executive functioning and memory may be due to sleep disturbances. Moreover, it is not clear whether a higher dose of glucocorticoid replacement, versus a lower dose may have an impact on cognition. The extent to which cognition impairment is described amongst this group seems to be mild and the effect on memory appears to be sleep-related or based on a propensity to developing hypoglycaemia. Studies exploring the relationship between neurocognition and hypoadrenalism have intrinsic weaknesses. It seems that there are no gross neuropsychological impairments in patients with adrenal insufficiency receiving replacement therapy, which to some degree is reassuring. Additionally, the various dosing regimens do not significantly impact negatively on cognition. Despite this, it is of critical importance for the clinician to screen patients with adrenal insufficiency for depression, which may manifest as cognitive impairment.

As treating Endocrinologists, we should remain sensitive to the subtle and overt cognitive derangements associated with serious endocrinological conditions and where possible enlist psychoeducation to address these deficits. Through greater awareness, it is hoped that rigorous options for addressing cognition in our group of patients will be revealed. The treating endocrinologist should be aware of the cognitive impact that may be associated with Cushing’s syndrome and adrenal insufficiency. An area that an endocrinologist should consider is the cognitive well-being of the patients and entertain the possibility of psychoeducation, with a view to enhancing patients’ ability to manage stress when encountering patients with adrenal insufficiency, Cushing’s syndrome, depression and dementia.

## Author contributions

IR: Writing – original draft, Writing – review & editing. EH: Writing – review & editing. MH: Writing – original draft, Writing – review & editing.

